# 5-Bromo-2-chloro­pyrimidin-4-amine

**DOI:** 10.1107/S1600536813007228

**Published:** 2013-03-23

**Authors:** Mohan Kumar, C. Mallikarjunaswamy, M. A. Sridhar, D. G. Bhadregowda, Kamini Kapoor, Vivek K. Gupta, Rajni Kant

**Affiliations:** a Department of Studies in Physics, Manasagangotri, University of Mysore, Mysore 570 006, India; b Department of Chemistry, Yuvarajas College, University of Mysore, Mysore 570 005, India; c X-ray Crystallography Laboratory, Post-Graduate Department of Physics & Electronics, University of Jammu, Jammu Tawi 180 006, India

## Abstract

In the title compound, C_4_H_3_BrClN_3_, the pyrimidine ring is essentially planar (r.m.s. deviation from the plane = 0.087 Å). In the crystal, pairs of N—H⋯N hydrogen bonds connect the mol­ecules into inversion dimers; these are connected by further N—H⋯N hydrogen bonds into a two-dimensional framework parallel to the *bc* plane.

## Related literature

For background to pyrimidine derivatives, see: Yu *et al.* (2007 ▶). For related structures, see: van Albada *et al.* (2012 ▶); Yang *et al.* (2012 ▶).
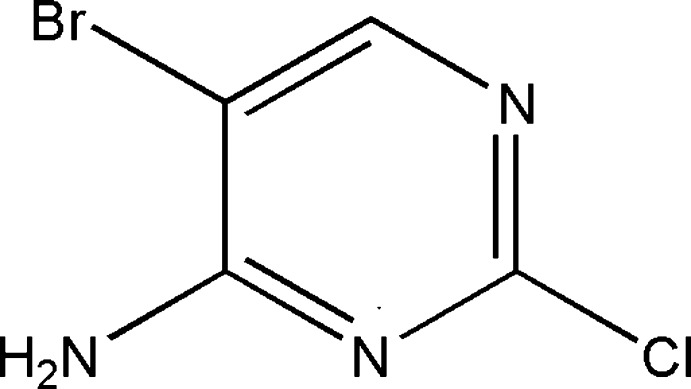


## Experimental

### 

#### Crystal data

C_4_H_3_BrClN_3_
*M* *_r_* = 208.45
Monoclinic, 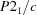

*a* = 6.0297 (1) Å
*b* = 8.1542 (2) Å
*c* = 13.4163 (3) Å
β = 90.491 (2)°
*V* = 659.62 (2) Å^3^
*Z* = 4
Mo *K*α radiation
μ = 6.54 mm^−1^
*T* = 293 K
0.3 × 0.2 × 0.1 mm

#### Data collection

Oxford Diffraction Xcalibur Sapphire3 diffractometer
Absorption correction: multi-scan (*CrysAlis PRO*; Oxford Diffraction, 2010 ▶) *T*_min_ = 0.306, *T*_max_ = 1.000
43395 measured reflections
1297 independent reflections
1164 reflections with *I* > 2σ(*I*)
*R*_int_ = 0.046

#### Refinement

*R*[*F* ^2^ > 2σ(*F* ^2^)] = 0.024
*wR*(*F* ^2^) = 0.058
*S* = 1.10
1297 reflections
90 parameters
H atoms treated by a mixture of independent and constrained refinement
Δρ_max_ = 0.33 e Å^−3^
Δρ_min_ = −0.28 e Å^−3^

### 

Data collection: *CrysAlis PRO* (Oxford Diffraction, 2010 ▶); cell refinement: *CrysAlis PRO*; data reduction: *CrysAlis RED* (Oxford Diffraction, 2010 ▶); program(s) used to solve structure: *SHELXS97* (Sheldrick, 2008 ▶); program(s) used to refine structure: *SHELXL97* (Sheldrick, 2008 ▶); molecular graphics: *ORTEP-3 for Windows* (Farrugia, 2012 ▶); software used to prepare material for publication: *PLATON* (Spek, 2009 ▶).

## Supplementary Material

Crystal structure: contains datablock(s) I, global. DOI: 10.1107/S1600536813007228/gk2560sup1.cif

Structure factors: contains datablock(s) I. DOI: 10.1107/S1600536813007228/gk2560Isup2.hkl

Supplementary material file. DOI: 10.1107/S1600536813007228/gk2560Isup3.cml

Additional supplementary materials:  crystallographic information; 3D view; checkCIF report

## Figures and Tables

**Table 1:** Hydrogen-bond geometry (Å, °)

*D*—H⋯*A*	*D*—H	H⋯*A*	*D*⋯*A*	*D*—H⋯*A*
N7—H71⋯N1^i^	0.78 (3)	2.38 (3)	3.087 (3)	153 (3)
N7—H72⋯N3^ii^	0.91 (4)	2.19 (4)	3.088 (3)	171 (3)

Symmetry codes: (i) 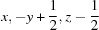; (ii) 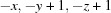.

**Fig. 1.**
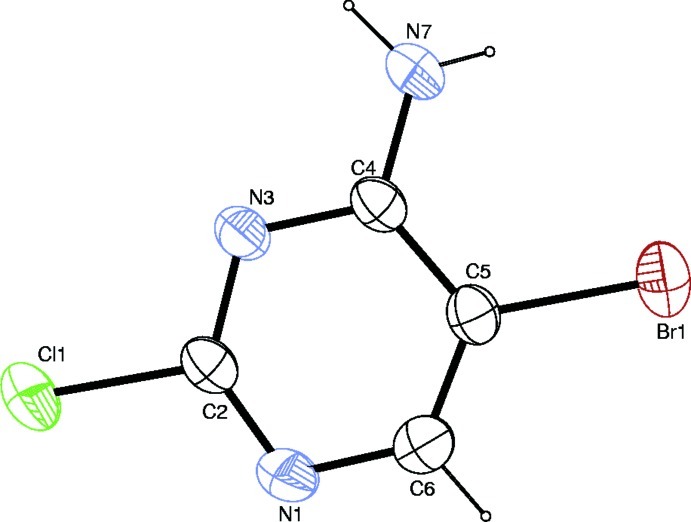
ORTEP view of the molecule with the atom-labeling scheme. The displacement ellipsoids are drawn at the 40% probability level. H atoms are shown as small spheres of arbitrary radii.

**Fig. 2.**
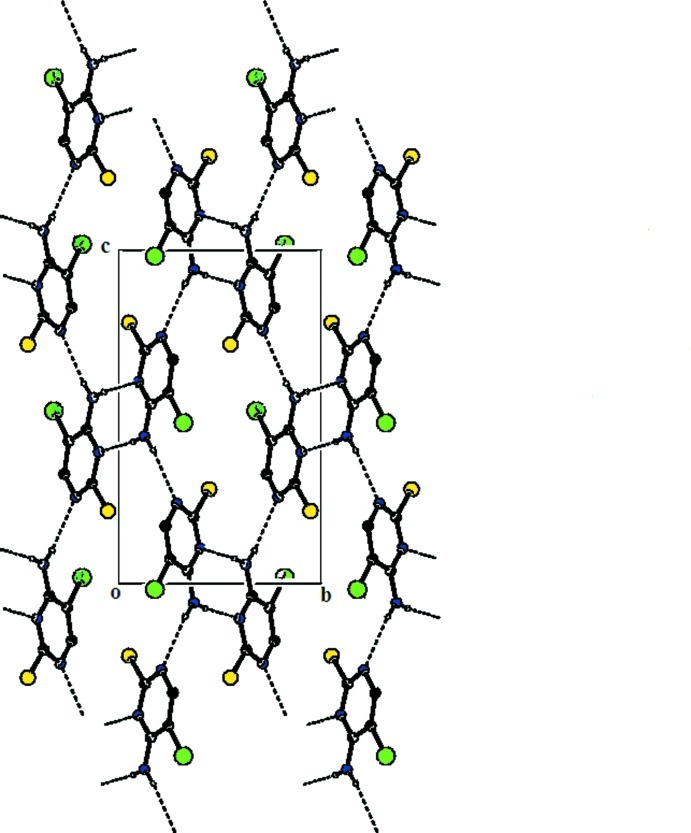
A molecular packing view of the title compound down the a axis, showing intermolecular interactions. The dotted lines show intermolecular N—H···N hydrogen bonds.

## supplementary crystallographic information

### Comment

Some derivatives of pyrimidine are important chemical materials (Yu *et al.*, 2007). Here in this article, the preparation and crystal structure of the title compound is presented. Bond lengths and angles in the title compound (Fig. 1) are comparable with the similar crystal structures (van Albada *et al.*, 2012; Yang *et al.*, 2012). The pyrimidine ring is essentially planar (r.m.s. deviation from the plane 0.087 Å). The atoms Br,Cl and N7 are coplanar with the pyrimidine ring. In the crystal, molecules are linked into dimers by N7—H72···N3 hydrogen bonds and these dimers are further connected by N7—H71···N1 hydrogen bonds, forming two dimensional supramolecular network in the *bc* plane (Fig.2, (Table 2).

### Experimental

To a solution of stannous chloride dihydrate (2.8 ml, 0.012 mole) in hydrochloric acid (30 ml) cooled to 273K, 5-bromo-2-chloro-4-nitropyrimidine (2 g, 0.0083 mole) was added in portions while the suspension was vigorously stirred for 6 hrs. The mixture was then poured onto crushed ice, made alkaline with solid sodium hydroxide, and extracted three times with ethyl acetate (100 ml). The combined organic phase was dried over anhydrous sodium sulfate and the filtrate was evaporated to dryness. The compound was purified by successive recrystallization from acetonitrile (yield 90%, m. p. 460–461 K).

### Refinement

The N-bound H atoms were located in a difference Fourier map and freely refined. All other H atoms were positioned geometrically and were treated as riding on their parent C atoms, with C—H distances of 0.93 Å and with *U*_iso_(H) = 1.2*U*_eq_(C).

### Crystal data

**Table:**

C_4_H_3_BrClN_3_	*F*(000) = 400
*M**_r_* = 208.45	*D*_x_ = 2.099 Mg m^−^^3^
Monoclinic, *P*2_1_/*c*	Mo *K*α radiation, λ = 0.71073 Å
Hall symbol: -P 2ybc	Cell parameters from 20319 reflections
*a* = 6.0297 (1) Å	θ = 3.7–29.0°
*b* = 8.1542 (2) Å	µ = 6.54 mm^−^^1^
*c* = 13.4163 (3) Å	*T* = 293 K
β = 90.491 (2)°	Block, white
*V* = 659.62 (2) Å^3^	0.3 × 0.2 × 0.1 mm
*Z* = 4	

### Data collection

**Table:**

Oxford Diffraction Xcalibur Sapphire3 diffractometer	1297 independent reflections
Radiation source: fine-focus sealed tube	1164 reflections with *I* > 2σ(*I*)
Graphite monochromator	*R*_int_ = 0.046
Detector resolution: 16.1049 pixels mm^-1^	θ_max_ = 26.0°, θ_min_ = 3.9°
ω scans	*h* = −7→7
Absorption correction: multi-scan (*CrysAlis PRO*; Oxford Diffraction, 2010)	*k* = −10→10
*T*_min_ = 0.306, *T*_max_ = 1.000	*l* = −16→16
43395 measured reflections	

### Refinement

**Table:**

Refinement on *F*^2^	Primary atom site location: structure-invariant direct methods
Least-squares matrix: full	Secondary atom site location: difference Fourier map
*R*[*F*^2^ > 2σ(*F*^2^)] = 0.024	Hydrogen site location: inferred from neighbouring sites
*wR*(*F*^2^) = 0.058	H atoms treated by a mixture of independent and constrained refinement
*S* = 1.10	*w* = 1/[σ^2^(*F*_o_^2^) + (0.0275*P*)^2^ + 0.4995*P*] where *P* = (*F*_o_^2^ + 2*F*_c_^2^)/3
1297 reflections	(Δ/σ)_max_ < 0.001
90 parameters	Δρ_max_ = 0.33 e Å^−^^3^
0 restraints	Δρ_min_ = −0.28 e Å^−^^3^

### Special details

**Table:**

Experimental. *CrysAlis PRO*, Oxford Diffraction Ltd., Version 1.171.34.40 (release 27–08-2010 CrysAlis171. NET) (compiled Aug 27 2010,11:50:40) Empirical absorption correction using spherical harmonics, implemented in SCALE3 ABSPACK scaling algorithm.
Geometry. All esds (except the esd in the dihedral angle between two l.s. planes) are estimated using the full covariance matrix. The cell esds are taken into account individually in the estimation of esds in distances, angles and torsion angles; correlations between esds in cell parameters are only used when they are defined by crystal symmetry. An approximate (isotropic) treatment of cell esds is used for estimating esds involving l.s. planes.
Refinement. Refinement of *F*^2^ against ALL reflections. The weighted *R*-factor *wR* and goodness of fit *S* are based on *F*^2^, conventional *R*-factors *R* are based on *F*, with *F* set to zero for negative *F*^2^. The threshold expression of *F*^2^ > σ(*F*^2^) is used only for calculating *R*-factors(gt) *etc*. and is not relevant to the choice of reflections for refinement. *R*-factors based on *F*^2^ are statistically about twice as large as those based on *F*, and *R*- factors based on ALL data will be even larger.

### Fractional atomic coordinates and isotropic or equivalent isotropic displacement parameters (Å^2^)

**Table:**

	*x*	*y*	*z*	*U*_iso_*/*U*_eq_	
Br1	0.69350 (4)	0.17972 (4)	0.48221 (2)	0.04520 (12)	
Cl1	−0.00058 (12)	0.44749 (10)	0.78250 (5)	0.04662 (19)	
N1	0.3588 (4)	0.2880 (3)	0.74005 (15)	0.0397 (5)	
C2	0.1934 (4)	0.3694 (3)	0.69923 (17)	0.0302 (5)	
N3	0.1489 (3)	0.4025 (3)	0.60525 (14)	0.0310 (4)	
C4	0.2921 (4)	0.3417 (3)	0.53797 (17)	0.0293 (5)	
C5	0.4802 (4)	0.2544 (3)	0.57261 (17)	0.0308 (5)	
C6	0.5050 (4)	0.2317 (4)	0.67197 (19)	0.0400 (6)	
H6	0.6288	0.1744	0.6947	0.048*	
N7	0.2498 (5)	0.3721 (3)	0.44228 (16)	0.0413 (6)	
H71	0.317 (5)	0.329 (3)	0.401 (2)	0.037 (8)*	
H72	0.125 (6)	0.428 (4)	0.425 (3)	0.063 (10)*	

### Atomic displacement parameters (Å^2^)

**Table:**

	*U*^11^	*U*^22^	*U*^33^	*U*^12^	*U*^13^	*U*^23^
Br1	0.03877 (18)	0.05169 (19)	0.04536 (18)	0.00220 (12)	0.01297 (12)	−0.00698 (12)
Cl1	0.0474 (4)	0.0627 (5)	0.0300 (3)	0.0046 (3)	0.0130 (3)	−0.0023 (3)
N1	0.0393 (12)	0.0544 (14)	0.0254 (10)	0.0025 (10)	0.0014 (9)	0.0059 (9)
C2	0.0323 (13)	0.0332 (12)	0.0251 (11)	−0.0060 (10)	0.0038 (9)	−0.0011 (9)
N3	0.0335 (11)	0.0357 (11)	0.0238 (9)	−0.0003 (9)	0.0024 (8)	−0.0001 (8)
C4	0.0300 (12)	0.0331 (13)	0.0247 (11)	−0.0069 (10)	0.0022 (9)	−0.0016 (9)
C5	0.0303 (13)	0.0312 (12)	0.0309 (12)	−0.0037 (10)	0.0048 (10)	−0.0016 (10)
C6	0.0347 (14)	0.0483 (16)	0.0371 (13)	0.0052 (12)	−0.0010 (11)	0.0049 (12)
N7	0.0448 (14)	0.0569 (15)	0.0221 (11)	0.0080 (12)	0.0027 (10)	−0.0018 (10)

### Geometric parameters (Å, º)

**Table:**

Br1—C5	1.877 (2)	C4—N7	1.330 (3)
Cl1—C2	1.744 (2)	C4—C5	1.415 (3)
N1—C2	1.314 (3)	C5—C6	1.353 (3)
N1—C6	1.355 (3)	C6—H6	0.9300
C2—N3	1.315 (3)	N7—H71	0.78 (3)
N3—C4	1.349 (3)	N7—H72	0.91 (4)
			
C2—N1—C6	112.7 (2)	C6—C5—Br1	121.5 (2)
N1—C2—N3	130.6 (2)	C4—C5—Br1	120.20 (17)
N1—C2—Cl1	115.35 (18)	C5—C6—N1	123.4 (2)
N3—C2—Cl1	114.02 (18)	C5—C6—H6	118.3
C2—N3—C4	116.1 (2)	N1—C6—H6	118.3
N7—C4—N3	117.3 (2)	C4—N7—H71	121 (2)
N7—C4—C5	123.9 (2)	C4—N7—H72	119 (2)
N3—C4—C5	118.8 (2)	H71—N7—H72	119 (3)
C6—C5—C4	118.3 (2)		
			
C6—N1—C2—N3	0.5 (4)	N3—C4—C5—C6	1.9 (4)
C6—N1—C2—Cl1	−179.43 (19)	N7—C4—C5—Br1	2.1 (3)
N1—C2—N3—C4	1.4 (4)	N3—C4—C5—Br1	−175.96 (17)
Cl1—C2—N3—C4	−178.71 (17)	C4—C5—C6—N1	0.1 (4)
C2—N3—C4—N7	179.4 (2)	Br1—C5—C6—N1	177.9 (2)
C2—N3—C4—C5	−2.5 (3)	C2—N1—C6—C5	−1.2 (4)
N7—C4—C5—C6	179.9 (3)		

### Hydrogen-bond geometry (Å, º)

**Table:**

*D*—H···*A*	*D*—H	H···*A*	*D*···*A*	*D*—H···*A*
N7—H71···N1^i^	0.78 (3)	2.38 (3)	3.087 (3)	153 (3)
N7—H72···N3^ii^	0.91 (4)	2.19 (4)	3.088 (3)	171 (3)

Symmetry codes: (i) *x*, −*y*+1/2, *z*−1/2; (ii) −*x*, −*y*+1, −*z*+1.
